# Targeting metabolic sensing switch GPR84 on macrophages for cancer immunotherapy

**DOI:** 10.1007/s00262-023-03603-3

**Published:** 2024-02-13

**Authors:** Jianying Li, Anjun Ma, Ruohan Zhang, Yao Chen, Chelsea Bolyard, Bao Zhao, Cankun Wang, Thera Pich, Wantong Li, Nuo Sun, Qin Ma, Haitao Wen, Steven K. Clinton, William E. Carson, Zihai Li, Gang Xin

**Affiliations:** 1 Department of Microbiology and Immunology, Pelotonia Institute for Immuno-Oncology, The James Comprehensive Cancer Center, The Ohio State University, 460 W 12th Ave, Columbus, OH 43210 USA; 2 Department of Microbial Infection and Immunity, The Ohio State University College of Medicine, Columbus, OH USA; 3 Department of Biomedical Informatics, The Ohio State University, Columbus, OH 43210 USA; 4 Dorothy M. Davis Heart and Lung Research Institute, The Ohio State University Wexner Medical Center, Columbus, OH USA; 5 Department of Physiology and Cell Biology, College of Medicine, The Ohio State University Wexner Medical Center, Columbus, OH USA; 6 Shanghai Institute of Immunology, Department of Immunology and Microbiology, Shanghai Jiao Tong University School of Medicine, Shanghai, China; 7 Department of Urology, The Ohio State University College of Medicine, Columbus, OH USA; 8 Department of Surgery, The Ohio State University College of Medicine, Columbus, OH USA

**Keywords:** Immunometabolsim, Cancer immunotherapy, Tumor-associated macrophage

## Abstract

**Introduction:**

As one of the major components of the tumor microenvironment, tumor-associated macrophages (TAMs) possess profound inhibitory activity against T cells and facilitate tumor escape from immune checkpoint blockade therapy. Converting this pro-tumorigenic toward the anti-tumorigenic phenotype thus is an important strategy for enhancing adaptive immunity against cancer. However, a plethora of mechanisms have been described for pro-tumorigenic differentiation in cancer, metabolic switches to program the anti-tumorigenic property of TAMs are elusive.

**Materials and methods:**

From an unbiased analysis of single-cell transcriptome data from multiple tumor models, we discovered that anti-tumorigenic TAMs uniquely express elevated levels of a specific fatty acid receptor, G-protein-coupled receptor 84 (GPR84). Genetic ablation of GPR84 in mice leads to impaired pro-inflammatory polarization of macrophages, while enhancing their anti-inflammatory phenotype. By contrast, GPR84 activation by its agonist, 6-n-octylaminouracil (6-OAU), potentiates pro-inflammatory phenotype via the enhanced STAT1 pathway. Moreover, 6-OAU treatment significantly retards tumor growth and increases the anti-tumor efficacy of anti-PD-1 therapy.

**Conclusion:**

Overall, we report a previously unappreciated fatty acid receptor, GPR84, that serves as an important metabolic sensing switch for orchestrating anti-tumorigenic macrophage polarization. Pharmacological agonists of GPR84 hold promise to reshape and reverse the immunosuppressive TME, and thereby restore responsiveness of cancer to overcome resistance to immune checkpoint blockade.

**Supplementary Information:**

The online version contains supplementary material available at 10.1007/s00262-023-03603-3.

## Introduction

Tumor-associated macrophages (TAMs) pose significant challenges for successful immunotherapy. The power of immune checkpoint blockade (ICB) has been established in more than 22 types of cancer [1]. ICB can achieve durable, complete tumor regression in multiple types of malignancy including colon cancers [2, 3]. However, less than 20% of patients with solid tumors overall benefit from this revolutionary therapy due to multiple resistance mechanisms, including the immunosuppressive tumor microenvironment (TME) [4]. Recent studies on the TME have revealed that pro-tumorigenic macrophages are one of the most abundant immunosuppressive cells, with a profound ability to limit the anti-tumor response [5]. TAMs comprise a heterogeneous population that can either help the tumor escape from immune attack (e.g., pro-tumorigenic) or help CD8 T cells eliminate the tumor (e.g., anti-tumorigenic) [5, 6]. The anti-tumorigenic TAMs are associated with immune activation, a favorable response to ICB in various cancer patients [7]. These pro-inflammatory macrophages produce immune-stimulating molecules, including inducible nitric oxide synthase (iNOS), tumor necrosis factor-alpha (TNFα), and CD86 [6, 8]. However, the majority of macrophages from established solid tumors typically exhibit an anti-inflammatory phenotype that supports tumor growth and abolishes immune surveillance [9, 10]. These pro-tumorigenic TAMs can produce an array of inhibitory molecules such as Arginase 1 (Arg1) and transforming growth factor-beta (TGFβ) to exert an immunosuppressive function and hinder the activation of multiple immune cells [5, 11]. This polarization between anti- and pro-tumorigenic phenotypes represents the plasticity of TAMs, and is controlled tightly by TME-derived factors. Based on this plasticity and enrichment of pro-tumorigenic TAMs in ICB-resistant patients, reprogramming TAMs toward the anti-tumorigenic phenotype is an attractive strategy for boosting immunotherapy.

Despite the prospect of boosting immunotherapy, macrophage-based therapies achieve limited success in improving clinical outcomes largely due to poor specificity [12–14]. The majority of current approaches are designed to systemically deplete or reprogram macrophages, and usually result in suboptimal efficacy against tumor-infiltrating macrophages [15]. Another challenge is unwanted side effects due to the wide tissue distribution and complex involvement of macrophages in various pathological conditions [16–18]. The  systemic reduction of macrophages may leave cancer patients more susceptible to infectious diseases and may disrupt immune homeostasis and organ function [18–20]. For example, inhibition of CSF1R signaling can dampen M2-like F4/80^high^ MHC-II^low^ TAM and suppress tumor growth [21–23]. However, this approach may have a detrimental effect on cardiac function and tissue repair during resolution from inflammation [24–26]. On the other hand, endorsing M1-like pro-inflammatory macrophages could lead to systemic inflammation and tissue damage [12, 14]. To overcome these issues, a novel tumor-specific factor that governs TAM differentiation is essential for designing safer and more effective TAM-targeted therapeutics for cancer patients.

As one of the hallmarks of malignancy, cancer cells alter their metabolic profile to engage in de novo fatty acid synthesis. Together with the accumulation of adipocytes and adipocyte-like fibroblasts in TME, tumor cells create a fatty acid-enriched environment [27]. The fatty acids have been extensively studied as an energy source to regulate the function of TAMs, however, the aspect of fatty acids serving as a signaling molecule remains obscure. Increasing evidence reveals fatty acid can activate their surface receptors and mediate signaling to control various physiological functions, including metabolic regulation and inflammatory response [28, 29], but has not been adequately explored in the context of anti-tumor immunity. Through analysis of single-cell RNA sequencing (scRNA-seq) datasets from several clinical studies, we identified one fatty acid sensor, G-protein-coupled receptor 84 (GPR84) is uniquely enriched in tumor-infiltrating myeloid cells but not abundant in other immune cells nor in normal tissue. Therefore, targeting GPR84 may have limited impact on tissue resident macrophages with fewer side effects. As a newly discovered receptor, GPR84 has been characterized as a medium chain fatty acid receptor and a critical regulator of phagocytosis and pro-inflammatory response in pulmonary fibrosis, ulcerative colitis, and neuroinflammation [30]. Although the underlying molecular mechanism is not well defined, several studies identify that GPR84 activation reduces cyclic adenosine monophosphate (cAMP) to regulate the downstream pro-inflammatory signaling [31]. However, the role of GPR84 in regulating macrophage polarization in tumors remains largely unknown. Our observation is the first to reveal that conditional deletion of *Gpr84* in myeloid cells promotes polarization of pro-tumorigenic TAMs with a mild effect on the proportion of myeloid-derived suppressor cells (MDSCs). As a result, GPR84 deficiency accelerates tumor growth with augmented immunosuppressive TAMs and dampened CD8 T cell function. Furthermore, the GPR84 agonist 6-OAU promotes pro-inflammatory polarization in macrophages without impacting MDSC differentiation in vitro. Mechanistically, we identify that GPR84 activation promotes a pro-inflammatory phenotype via augmenting STAT1 pathway for the first time. Consequently, activating GPR84 pharmacologically not just produces profound anti-tumor immunity but also synergizes with PD-1 blockade. These data build a strong premise for the role of GPR84 as a metabolic signaling checkpoint in TAM polarization. Altogether, these findings suggest that GPR84 endorses anti-tumor immunity by inducing pro-inflammatory TAMs via the STAT1 pathway and that targeting this pathway can improve the efficacy of ICB.

## Results

### GPR84 expression in TAMs correlates with antitumor immune response

**Fig. 1:**
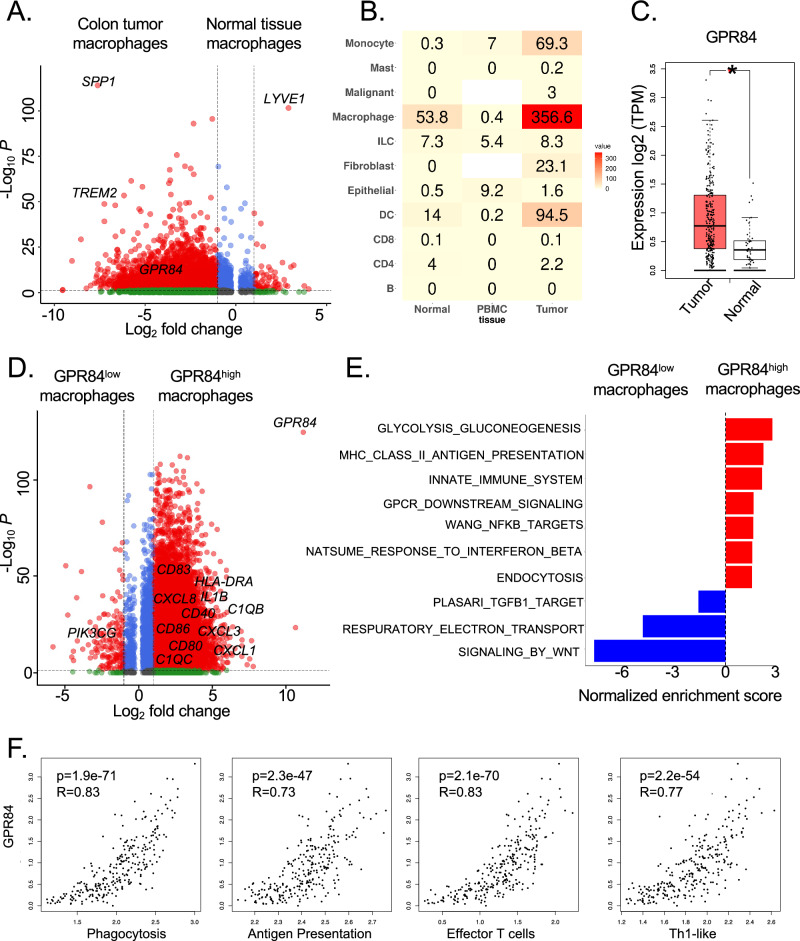
Analysis of single-cell and bulk transcriptome of multiple cancer types reveals the preferential expression of GPR84 in TAMs. **A** The scRNA-seq dataset from colon tumor and adjacent normal tissues were re-analyzed. The volcano plot demonstrated gene transcripts specifically enriched in myeloid cells from colon tumor (*TREM2*, *GPR84* and *SPP1*) and adjacent normal tissues (*LYVE1*). **B** Expression of *GPR84* in Myeloid, ILC, Epithelial, CD8, CD4, and B cells in adjacent normal tissues, PBMC, and tumor tissue in this scRNA-seq dataset and tissue prevalence were estimated by Ro/e score. **C** The expression of *GPR84* from TCGA (Tumor tissue = 275) and GTEx (normal tissue = 41) were compared using web server GEPIA2. Statistical significance was determined using the Fisher exact test with a confidence level of 95%. *P < 0.05. **D** The tumor-infiltrating myeloid cells were re-analyzed from scRNA-seq dataset. The volcano plot shows differential gene expression in *GPR84*^high^ and *GPR84*^low^ myeloid cells in colon cancer. **E** Pathway analysis of differentially expressed genes by *GPR84*^high^ and *GPR84*^low^ myeloid cells. **F** GEPIA2 was used to analysis the correlation of *GPR84* and genes related to the phagocytosis (*MRC1*, *CD163*, *MERTK* and *C1QB*), antigen presentation (KEGG ANTIGEN PROCESSING AND PRESENTATION), effector T cells (*CX3CR1*, *FGFBP2* and *FCGR3A*), and Th1-like signature genes (*CXCL13, HAVCR2, IFNG, CXCR3, BHLHE40* and *CD4*) from TCGA dataset

To define key genes that are specifically upregulated by TAMs and involved in regulating anti-tumor immunity, we re-analyzed recent scRNA-seq datasets of immune cells from colon tumors, adjacent normal tissues and blood using their original cell annotations [32]. Differential expression analysis between macrophages from the tumor and normal tissue identified genes that are enriched specifically within TAMs (*TREM2*, *GPR84* and *SPP1*) and tissue-resident macrophages (*LYVE1*), which is consistent with previous observation (Fig. 1A) [32]. Among these tumor-enriched genes, fatty acid sensor, *GPR84*, is predominantly upregulated in the tumor-infiltrating macrophages cells, but barely detectable in adjacent benign tissues, peripheral blood mononuclear cells and other immune cells such as innate lymphoid cells (ILCs), T, and B cells (Fig.  1B and Supplemental Figure 1A) [32]. To extend this observation to other tumor types, we expanded our analysis on tumor-infiltrating immune cells from 10 different scRNA-seq datasets and obtained similar observations in various cancer types including bladder and breast cancer (Supplemental 1B). To confirm this finding in a larger cohort, we used the web server, GEPIA2 [33] to interrogate The Cancer Genome Atlas (TCGA) human cancer and the Genotype Tissue-Expression (GTEx) database for *GPR84* expression, which exhibits significantly higher expression in colon tumors than normal tissues (Fig. 1C). Furthermore, similar expression profiles were also observed in other tumors including bladder and breast cancer (Supplemental 1C). To further confirm the correlation between *GPR84* and TAMs, our TCGA-based analysis demonstrated that the high expression levels of *GPR84* are strongly associated with macrophage signature markers (Supplemental 1D). Matching these clinical observations, our flow cytometry studies using the MC38 colon cancer model showed that the protein level of GPR84 is abundant in tumor samples, but significantly lower in spleen, bone marrow, and blood (Supplemental 1E). Within the tumor, the majority of GPR84 proteins (more than 90%) are restricted to MDSCs and macrophages, whereas only 5% of GPR84 is found in other immune cells (Supplemental 1F–H). All of these results suggest that the expression of GPR84 is limited to tumor-infiltrating myeloid cells.

Within the TAM subsets, the expression of *GPR84* is preferably found on the *C1QC*+ inflammatory TAMs with enhanced complement activation and antigen processing and presentation pathways (Supplemental 1I) [32]. To examine the role of GPR84 in TAMs, we next compared differentially expressed genes (DEGs) between *GPR84*^high^ and *GPR84*^low^ tumor-infiltrating macrophages from the same colon cancer scRNA-seq datasets [32]. Our integrative analysis revealed that genes related to anti-tumorigenic activity such as, *CD40*, *CD80*, *CD86*, *C1QC *and *HLA-DRA* were highly expressed in *GPR84*^high^ cells (Fig. 1D). Similar, gene set enrichment analysis revealed that *GPR84*^high^ macrophages exhibited several transcript signatures associated with immune activation phenotypes, such as the TNF signaling via NF-κB signaling, antigen presentation and Interferon Beta response (Fig. 1E). Because many studies highlighted the impact of TAMs on T cell function, we further explored the connection between GRP84 and anti-tumor T cell response. Our TCGA analysis confirmed the significant positive association between *GPR84* and antigen presentation signature genes (Fig. 1F). In addition, the abundance of *GPR84* is correlated with enhanced effector T cells and Th1-like signature genes (Fig. 1F). Together, these data suggest that GPR84 expression in TAMs positively correlates with an anti-tumorigenic immune phenotype.

### GPR84 expression is critical for macrophage polarization in vitro

**Fig. 2:**
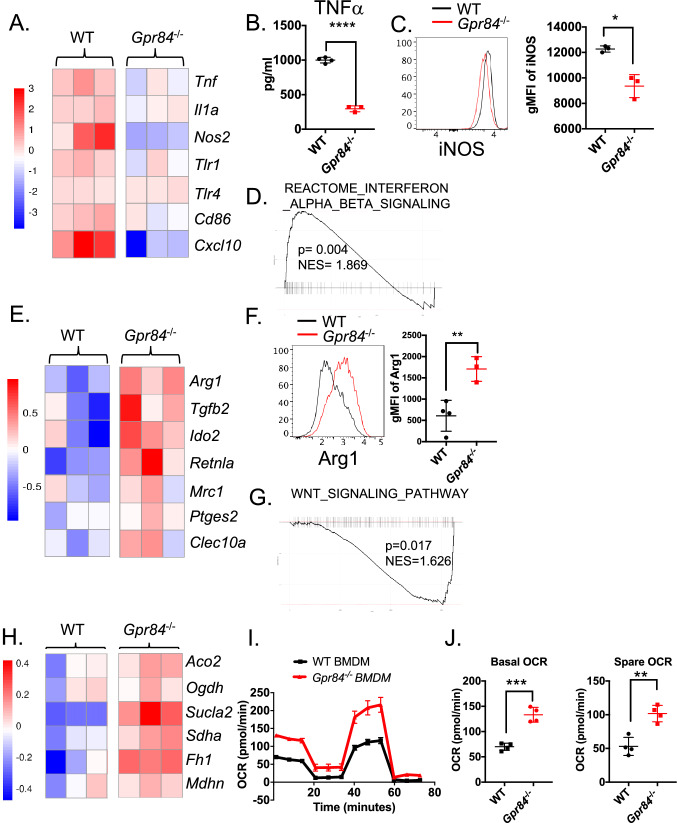
GPR84 deficiency favors anti-inflammatory macrophage polarization in vitro. **A** WT and *Gpr84*^−/−^ BMDMs were stimulated with LPS for 48 h and subjected for bulk RNA-seq. Heatmap shows the expression of pro-inflammatory genes in WT and *Gpr84*^−/−^ M(LPS) BMDMs. **B** The TNF level in the medium from WT and *Gpr84*^−/−^ M(LPS) BMDMs culture was measured by ELISA at the same time. **C** Representative flow histogram and summary data displaying the geometric Mean Fluorescent Intensity (gMFI) of iNOS on M(LPS) macrophage cells from WT and *Gpr84*^−/−^. **D** GSEA plots demonstrate the interferon alpha beta signaling pathways were negatively enriched in *Gpr84*^−/−^ M(LPS) BMDMs. **E** Bulk RNAseq was performed on WT and *Gpr84*^−/−^ BMDMs treated with IL-4 for 48 h. Heat map representing genes differentially expressed between WT and *Gpr84*^−/−^ M(IL-4) BMDMs. **F** Representative flow histogram and summary data display the gMFI of Arg1 in WT and *Gpr84*^−/−^ groups. **G** GSEA plots depict the WNT signal gene signatures positively enriched in *Gpr84*^−/−^ M(IL-4) BMDMs. **H** Heatmap showing gene expression of TCA-related enzymes. **I** Line graphs depicting the oxygen consumption rate (OCR) of WT and *Gpr84*^−/−^ M(IL-4) BMDMs in response to the Mito Stress assay. **J** Bar graphs quantifying basal respiratory capacity (BRC) and spare respiratory capacity (SRC) in WT and *Gpr84*^−/−^ M(IL-4) BMDMs (*n* = 4). Data are expressed as mean ± SEM and *n* = 3. *P < 0.05, **P < 0.01, ***P < 0.001, ****P < 0.0001

We sought to dissect the role of GPR84 in macrophage differentiation. To exclude the possible impact of GPR84 on other immune cells, we activated CD4 and CD8 T cells via anti-CD3/CD28 stimulation and observed no activation deficiency in *Gpr84*^*−*/*−*^ mice (Supplemental 2A, B). Wild-type (WT) and *Gpr84*^*−*/*−*^ bone marrow-derived MDSCs and dendritic cells exhibited similar levels of differentiation markers such as Gr-1, Arg1 and CD11c, respectively (Supplemental 2C–E). Furthermore, bone marrow-derived macrophages (BMDMs) generated from *Gpr84*^*−*/*−*^ mice exhibited comparable level of surface maker (CD11b and F4/80) and transcripts related to macrophage phenotype and differentiation as WT BMDMs, suggesting no impact of GPR84 on macrophage development (Supplemental 2F–H). We next examined the function of GPR84 in pro or anti-inflammatory activation of macrophages [31]. In response to the stimulation with lipopolysaccharide (LPS), *Gpr84*^*−*/*−*^ BMDMs expressed significantly less pro-inflammatory genes, including *Tnf, Il1a, Nos2*, *Cd86* and *Cxcl10* (Fig. 2A). Consistent with transcript levels, proteins of inflammatory mediators such as TNF-α and iNOS, was markedly reduced in the setting of *Gpr84*^*−*/*−*^ BMDMs compared to the WT BMDMs (Fig. 2B, C). Furthermore, *Gpr84*^*−*/*−*^ M(LPS) BMDMs contained significantly less signature genes related to Interferon Alpha/Beta signaling (Fig. 2D). Conversely, in response to IL-4-stimulation, *Gpr84*^*−*/*−*^ BMDMs exhibited a substantial upregulation of immunosuppressive signature genes such as *Arg1*, *Tgfb3* and *Retnla* (Fig. 2E). Consistently, the protein level of Arg1 also increased significantly in *Gpr84*^*−*/*−*^ M(IL-4)-BMDMs (Fig. 2F). Gene set enrichment analysis revealed that the gene signature associated with WNT signaling pathways is highly enriched in *Gpr84*^*−*/*−*^ M(IL-4)-BMDMs (Fig. 2G). In addition, the genes encoding enzymes in the mitochondrial TCA cycle are enhanced in *Gpr84*^*−*/*−*^ M(IL-4)-BMDMs (Fig. 2H). To determine whether these transcriptional alternations led to any metabolic changes, we measured oxygen consumption rates (OCR) using a Seahorse extracellular flux analyzer. Notably, *Gpr84*^*−*/*−*^ BMDMs showed significantly higher basal respiratory capacity (BRC) and higher spare respiratory capacity (SRC) than WT BMDMs (Fig. 2I, J).Collectively, these data suggest that lack of GPR84 antagonize pro-inflammatory macrophage polarization while promoting anti-inflammatory macrophage phenotype.

### GPR84 deficiency promote pro-tumorigenic TAMs in vivo

**Fig. 3:**
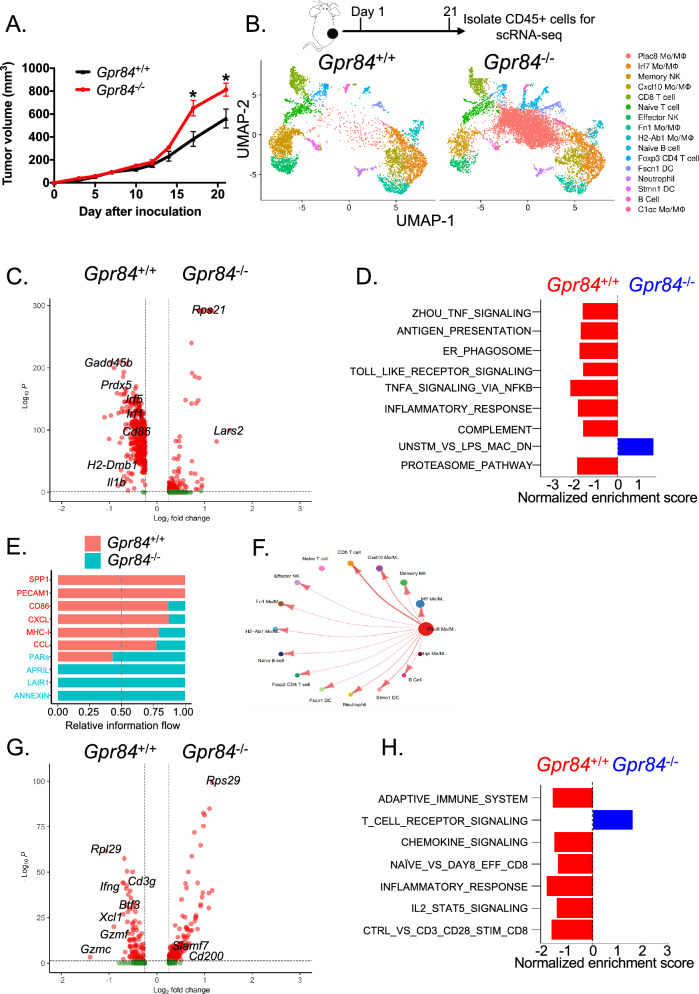
*Gpr84*^−/−^ mice exhibits reduced anti-tumorigenic TAMs in vivo. **A** *Gpr84*^−/−^ (*n* = 5) and *Gpr84*^+/+^ (*n* = 5) mice were inoculated with MC38 and tumor growth was monitored and compared by multiple *t*-test. **B** Experimental design of scRNA-seq. The tumor-infiltrating CD45^+^ cells were sorted from *Gpr84*^−/−^ (*n* = 4) and *Gpr84*^+/+^ (*n* = 4) mice at 21 days past tumor inculcation and pooled together. After initial analysis, 16 immune-cell clusters from both groups were annotated and shown in UMAP. **C** A volcano plot showing differential gene expression in mono/macrophage clusters from *Gpr84*^+/+^ and *Gpr84*^−/−^ tumors. **D** Pathways with significantly different activities mono/macrophage clusters between *Gpr84*^−/−^ and *Gpr84*^+/+^. **E** The Cell Chat analysis revealed the overall signaling strength weights calculated as the product of the average receptor-ligand expression between *Gpr84*^+/+^ and *Gpr84*^−/−^. **F** The circle plot shows the cell communication from *Plac8* Mo/Macrophage cluster to other clusters and edge thickness indicates the sum of weighted paths between populations **G** The volcano plot demonstrates differential gene expression in CD8 cells from *Gpr84*^+/+^ and *Gpr84*^−/−^. **H** Pathways with significantly different activities in CD8 cell clusters. *P < 0.05

We next sought to determine the in vivo role of GPR84 in anti-tumor immunity using the MC38 colon carcinoma model. In this model, pro-tumorigenic macrophages can suppress anti-tumor immunity and accelerate tumor progression. Mice lacking GPR84 exhibited more aggressive tumor growth than littermate control *Gpr84*^+/+^ (Fig. 3A). To investigate the transcriptional programs that underlie this difference, we performed scRNA-seq on tumor-infiltrating immune cells from *Gpr84*^+/+^ and *Gpr84*^*−*/*−*^ MC38 tumor-bearing mice on day 21 past inoculation (Fig. 3B). First, cell clusters were identified by comparison with the ImmGen database and assessment of known cell-type markers. Based on the level of various myeloid (*Adgre1* encodes F4/80, *Itgam* encodes CD11b, and *S100a9*) and lymphoid (*Cd4*, *Cd8b1*, *Ncr1*) markers, we delineated one CD8 T cell cluster, one CD4 T cell cluster, two B cell clusters, six mono/macrophage cell clusters, one neutrophil cluster, two DC clusters and two NK cell clusters (Fig. 3B and Supplemental 3A). We found that the absence of GPR84 results in a notable increase in one mono/macrophage cluster that displayed significant levels of Plac8 expression (Fig. 3B and Supplemental 3B). This subset has been linked to immunosuppressive activity [34] and lower survival rates for breast cancer patients [35]. Meanwhile, another mono/macrophage cluster with heightened FN1 levels, which is associated with pro-angiogenic TAMs and necessary for cancer metastasis [36], was amplified in the tumor-bearing mice lacking *Gpr84* (Fig. 3B and Supplemental 3B). These findings suggest that GPR84 is crucial in regulating the pro-tumorigenic macrophages. On the contrary, the deletion of GPR84 leads to reduction of *H2-Ab1* (MHCII) and *C1qc* expressing mono/macrophage clusters (Fig. 3B and Supplemental 3B), which related to anti-tumorigenic function [37]. Similarly, the mono/macrophage cluster with higher expression of *Cxcl10*, which is a key chemokine responsible for CD8 T cell recruitment in tumor, are reduced in in *Gpr84*^*−*/*−*^ mice (Fig. 3B and Supplemental 3B). Differential gene expression analyses within these six mono/macrophage clusters revealed that genes associated with immune activation (*Cd40*, *Cd86*, *Cxc10*, *C1qC* and *H2-DMb1*) were downregulated dramatically in *Gpr84*^*−*/*−*^ mice (Fig. 3C). In the same line, signature genes related to anti-tumoral phenotypes, such as TNF signaling, phagosome, complement and antigen presentation was disrupted significantly in *Gpr84*^*−*/*−*^ TAMs (Fig. 3D). Next, to delve more deeply into the influence of GPR84 on macrophage polarization, we employed the software package Monocle to construct the differentiation trajectories of TAMs [38] (Supplemental 3C). Overall, these results support that lack of GPR84 shifts TAM polarization toward a pro-tumoral phenotype.

To gain further insight about how this altered TAM polarization impact other immune cells, we use Cell Chat to delineate the potential cellular communication between clusters based on known ligand-receptor interactions [39]. The overall ligand-receptor communications involved antigen presentation (MHCI), costimulation (CD86) and chemotaxis (CCL and CXCL) were weaker in *Gpr84*^*−*/*−*^ mice than *Gpr84*^+*/*+^ (Fig. 3E and Supplemental 3D). On cellular level, the interaction between mono/macrophage and CD8 T cell clusters has higher score among other clusters (Fig. 3F and Supplemental 3E), implicating a potential role of TAMs involved in T cell activation and recruitment. Consistently, CD8 T cells from *Gpr84*^*−*/*−*^ expressed lower level of effector molecule *Gzmc* (Fig. 3G), with a reduced transcriptional signature of effector T cell and chemokine (Fig. 3H). Our analysis collectively implies that GPR84 is required to induce the polarization of immune activation TAMs to support effector CD8 T cells.

### *Gpr84* deficiency in macrophages promotes tumor growth and impedes CD8+ T cell function

**Fig. 4:**
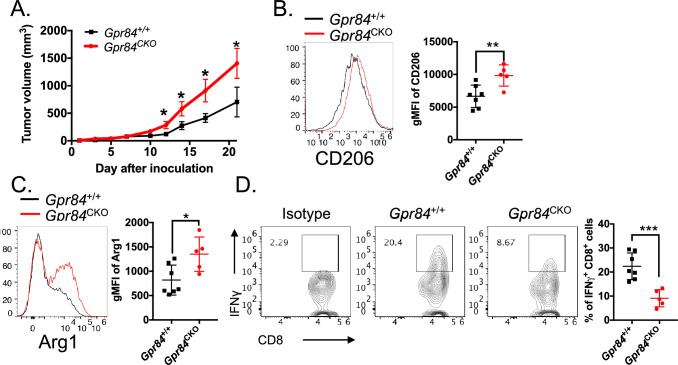
*Gpr84* deficiency in macrophage promotes immunosuppressive TAMs and abrogates anti-tumor response. **A** *Gpr84*^flox/flox^ *LysM*^Cre^ (*Gpr84*^CKO^) and littermate control *Gpr84*^flox/flox^ (*Gpr84*^+/+^) mice were inoculated with MC38 and tumor growth was monitored and compared by multiple *t*-test. **B**, **C** 21 days after inoculation, both group of mice were harvest for examining immune cells in TME. Flow cytometric expression of CD206 and Arg-1 protein on TAMs were measured by flow cytometry. **D** Ex vivo stimulation of tumor infiltrating T cells to measure the IFN*γ* producing CD8 T cells between *Gpr84*^CKO^ and *Gpr84*^+/+^. Data for all panels represent cumulative results from two independent experiments, *n* = 5–7 per group. *P < 0.05, **P < 0.01, ***P < 0.001

To determine the intrinsic role of GPR84 in TAMs, we have conditionally deleted *Gpr84* in the myeloid lineage by breeding the *Gpr84*^flox/flox^ mice with transgenic *LysM*^*Cre*^ mice, which express Cre recombinase under the control of the myeloid cell *Lysm* promoter. At homeostasis, this new strain *Gpr84*^*flox/flox*^* LysM*^*Cre*^ (*Gpr84*^CKO^) exhibited comparable immune profiles with littermate control *Gpr84*^*flox/flox*^ (*Gpr84*^+*/*+^) at the spleen, bone marrow and blood (Supplemental 4A–C). These two strains were inoculated subcutaneously with MC38 tumor. Intriguingly, tumor growth was significantly accelerated in *Gpr84*^CKO^ mice (Fig. 4A). This is largely associated with the fact that the CD11b^+^F4/80^+^CD64^+^Gr-1^low^ TAMs from *Gpr84*^CKO^ mice expressed higher levels of Arg1 and CD206 than littermate control (Fig. 4B, C), while lower level of CD86 (Supplemental 4D). Furthermore, the proportion of TAMs in tumors were only slight reduced in *Gpr84*^CKO^ mice (Supplemental 4E). To test whether there is a mechanistic link between TAM and CD8 T cell function, we sorted TAMs for a CD8 T cell co-culture suppression assay and found that the ability of *Gpr84*^CKO^ TAMs to inhibit T cell proliferation was significantly augmented (Supplemental 4F). As a result of enhanced immunosuppressive phenotype, we observed that IFNγ-producing CD8 T cells were significantly reduced in tumors from *Gpr84*^CKO^ mice (Fig. 4D). However, the percentages of CD4 and CD8 T cells among total CD45 + cells in tumor were comparable between *Gpr84*^CKO^ and littermate control (Supplemental 4G, H). To eliminate the possibility that these phenotypes are caused by the impact of GPR84 on other myeloid cells, our flow cytometry studies demonstrated that the proportion of tumor-infiltrating MDSCs were not affected by deletion of *Gpr84* (Supplemental 4I). Consistently, there is no significant difference in the immune cells, including CD4, CD8 T cells and DCs from the tumor or tumor-draining lymph nodes between *Gpr84*^+/+^ and *Gpr84*^CKO^(Supplemental 4J–L). Overall, these data suggest that loss of *Gpr84* in macrophages enhances tumor progression by favoring immunosuppressive TAMs and restricting CD8 T cells within the TME.

### GPR84 activation enhances pro-inflammatory macrophage via IFNβ/STAT1 pathway

**Fig. 5:**
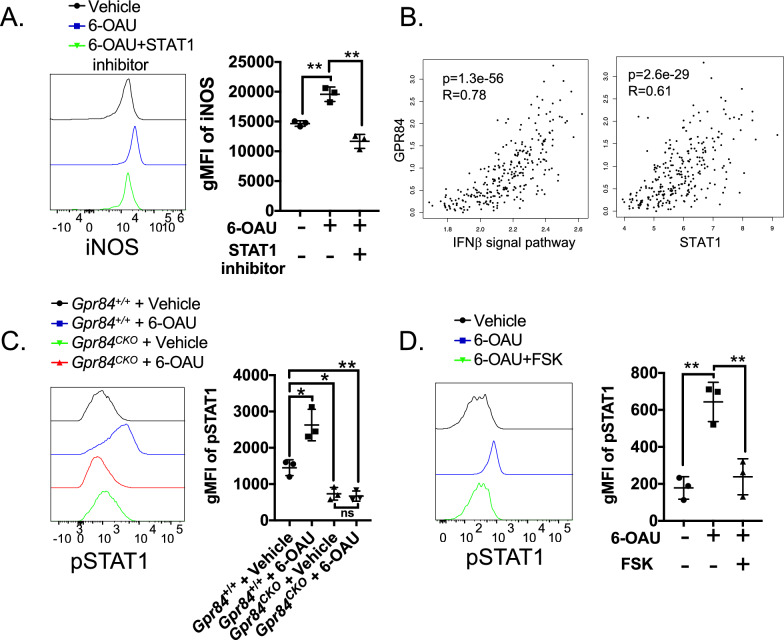
GPR84 activation shifts macrophages toward the pro-inflammatory phenotype. **A** The WT BMDMs were stimulated with LPS while receiving treatment with either vehicle (DMSO), 1 µM 6-OAU, or 1 µM 6-OAU + 1 µM fludarabine (STAT1 specific inhibitor) for 48 h. The level of iNOS was measured by flow cytometry. **B** GEPIA2 was used to analyze the correlation of GPR84 and genes related to the IFNβ signaling (HECKER_IFNB1_TARGETS) and STAT1. **C** The *Gpr84*^CKO^ and littermate control *Gpr84*^+/+^ BMDMs were treated with either vehicle (DMSO) or 1 µM 6-OAU. The level of pSTAT1 was measured by flow cytometry. **D** The WT M(LPS) BMDMs were treated with either vehicle (DMSO), 1 µM 6-OAU, or 1 µM 6-OAU + 25 µM forskolin (FSK) for 48 h and phospho-flow cytometry was used to detect pSTAT1. *P < 0.05, **P < 0.01, *n* = 3

Next, we sought to determine whether activation of GPR84 fosters a pro-inflammatory macrophage phenotype and found that GPR84 agonist 6-OAU can potentiate iNOS expression (Fig. 5A). Our bioinformatics analysis revealed that response to IFN beta is enriched in GPR84^high^ TAMs (Fig. 1E) and abolished in *Gpr84*^*−*/*−*^ BMDMs (Fig. 2D), which suggests a role of IFNβ in GPR84-mediated macrophage polarization. Similarly, TCGA analysis confirmed that  the expression of GPR84 was significantly correlated with IFNβ signaling signature and STAT1 (Fig. 5B). Selective inhibition of STAT1 can abolish the heightened iNOS expression in response to 6-OAU (Fig. 5A). Since IFNβ primarily signals through the STAT1 pathway [40], the phosphorylation of STAT1 was measured by flow cytometry to assay the activity of IFNβ signaling. Consistently, we found that the 6-OAU treatment significantly augmented the IFNβ pathway (Fig. 5C). This 6-OAU-induced STAT1 activity was absent in *Gpr84*^CKO^ BMDMs (Fig. 5C). Thus, we sought to determine the underlying mechanisms of GPR84-mediated STAT1 activation. As one of the new members of the Gαi family of GPCRs, the downstream pathway of GPR84 in macrophages is unclear. However, it is well-known that Gαi proteins, such as GPR84, typically inhibit adenylyl cyclase activity resulting in decreased intracellular cyclic adenosine monophosphate (cAMP) levels [41], which in turn increases the activity of ERK signaling pathways [31]. Taken together the role of ERK on activation of IFNβ [42, 43], it is plausible that GPR84 augments the IFNβ/STAT1 pathway through a cAMP/ERK-dependent mechanism. In support of this idea, the 6-OAU-enhanced pSTAT1 can be abolished by treating forskolin (FSK), an activator of adenylyl cyclase that increases cAMP production or SCH772984, an ERK inhibitor (Fig. 5D and Supplemental 5A). To exclude the possible impact of GPR84 on other myeloid cells, we treated bone marrow-derived MDSCs with 6-OAU and discovered that GPR84 activation has no impact on their in vitro differentiation (Supplemental 5B). Overall, these findings suggest that cAMP-modulated STAT1 is a major target of the GPR84 signaling in macrophages.

### GPR84 activation enhances the ICB efficacy against cancer

**Fig. 6:**
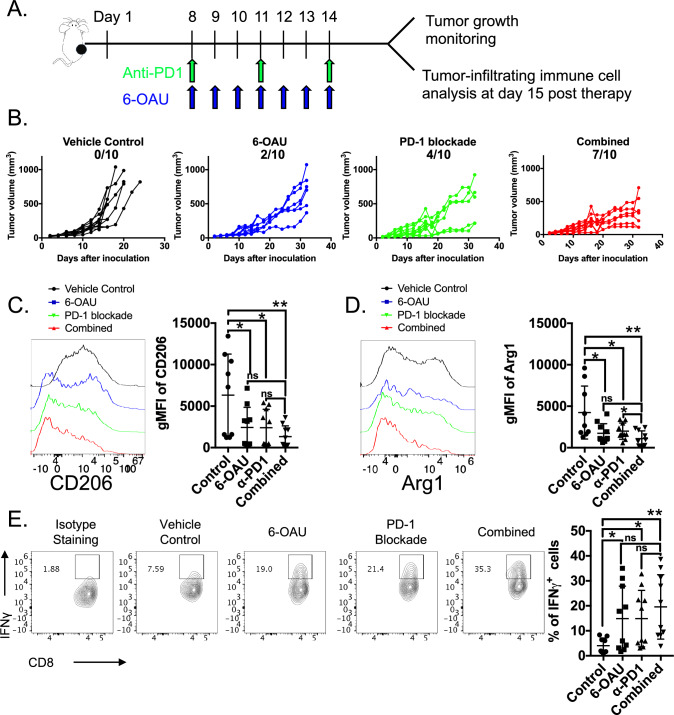
GPR84 activation synergizes with PD-1 blockade in cancer immunotherapy. **A** Schedule of experimental design. The MC38 tumor-bearing mice received the following treatments 8 days after tumor inoculation: vehicle control, 6-OAU (250 μg daily for 7 days), α-PD-1 mAb (200 μg on days 8, 11, and 14), or a combination of 6-OAU and α-PD-1 mAb. **B** The tumor growth curves for individual mice and responders are shown. **C** One day after the last treatment (day 15), all mice were harvest for flow cytometry analysis. The expression of CD206 was evaluated on CD45^+^ CD3^−^ CD11b^+^ Ly6g^−^ F4/80^+^ CD64^+^ macrophages in all groups. **D** Representative plot and quantification of Arg1 protein level on TAMs. **E** CD8 T cells were isolated from tumor and stimulated for evaluating cytokine production by flow cytometry as shown in representative and quantitative plots. Representative flow plots depicting the proportion and total number of IFN*γ*^+^ CD8 T cells. Tumor growth was measured using calipers for two weeks and plotted. Data for all panels represent cumulative results from two independent experiments, *n* = 9–10 per group. *P < 0.05, **P < 0.01, ****P < 0.0001

Based on this vital role of GPR84 on macrophage polarization in tumors, we reasoned that selective targeting of GPR84 would skew the anti-tumorigenic phenotype. To test this idea, we sought to investigate whether therapeutic activation of GPR84 promotes anti-tumor responses and synergizes with PD-1 blockade (Fig. 6A). Single-agent treatment of GPR84 with the activator 6-OAU delayed the tumor growth significantly (Fig. 6B). This effect is less likely due to direct cytotoxicity against cancer cells because in vitro 6-OAU treatment on two tumor cell lines failed to induce apoptosis (Supplemental 6A). More importantly, we found that co-administration of 6-OAU and anti-PD-1 monoclonal antibody induced more responders that experiencing tumor regression and higher survival rate than either single agent (Fig. 6B Supplemental 6B). These findings suggest that activation of GPR84 can augment an effective anti-tumor immune response.

We employed flow cytometry to assess the tumor-infiltrating lymphoid and myeloid populations one day after last treatment in order to delineate the mechanism of action of the 6-OAU-mediated anti-tumor efficacy (Fig. 6A). Based on our demonstration of the impact of 6-OAU on macrophage polarization, we hypothesized that GPR84 would elicit changes in the phenotype of TAMs. Consistently, we observed a reduced expression of CD206 on tumor-infiltrating macrophages, associated with pro-tumorigenic immunosuppressive TAMs, in both 6-OAU and combination treatment groups (Fig. 6C). These TAMs also exhibited substantial reduction on immune-regulatory molecules, such as Arg1 (Fig. 6D). We also observed a modest reduction in the frequency of CD11b^+^ Ly6c^high^ Ly6G^+^ MDSCs and moderate increase in CD4 and CD8 T cells (Supplemental 6C). Given the disrupted immunosuppressive phenotype of TAMs, the effector function of CD8 T cells, such as the production of IFNγ in combination-treated mice was scientifically enhanced compared to the vehicle control group (Fig. 6E). Taken together, our data suggest that GPR84 activation reshapes TAM-mediated immunosuppression to enhance cytotoxic T cell responses and overcome resistance to ICB.

## Discussion

Multiple recent studies have highlighted the promise of manipulating macrophages to boost anti-tumor immunity [5]. However, lack of TAM-specific targets leads to insufficient therapy and off-target side effects [12]. To address this issue, we conducted a comprehensive bioinformatic analysis and revealed that TAMs express a fatty acid sensor, GPR84, that is strongly associated with the anti-tumorigenic activity. Supporting this notion, our study revealed that ablation of GPR84 perturbs the pro-inflammatory, while favoring immunosuppressive anti-inflammatory polarization in macrophages. We further demonstrated that pharmacological activation of GPR84 reshapes TAMs to improve PD-1 blockade therapy in both colon and bladder cancer models. Our findings unravel an underexplored mechanism by which fatty acid signaling determines the pro/anti-tumorigenic activity of TAMs, and is critical for designing novel therapeutic strategies and improving ICB efficacy.

Although fatty acids have emerged as a critical regulator for macrophage polarization [12, 44, 45], most studies have focused on fatty acid oxidation without exploring the role of fatty acids as signaling molecules. In addition to fueling the energy supply, free fatty acids exert diverse functions by binding and activating fatty acid sensors [46]. One of such receptors, GPR84, is a specific receptor for medium-chain fatty acids of 9 to 14 carbon atoms in length. Previously, the expression of this fatty acid sensor has been found mainly in immune cells, including monocytes, macrophages and neutrophils [30]. Extending this observation, our analysis of 13 tumor types revealed that mRNA levels of GPR84 are restricted to macrophages from the tumor, but not adjacent benign and other tissues. In accordance with this observation, a growing body of evidence supports a regulatory role of GPR84 in macrophage-mediated inflammation in lung injury processes [47], kidney fibrosis [48], diabetes [49], and the central nervous system [50]. In addition, one recent in vitro CRISPR screen has identified GPR84 as an enhancer for phagocytosis of Ramos lymphoma cells [51]; which can be escaped by overexpression of adipocyte plasma membrane-associated protein (APMAP) [51]. Despite this in vitro study on hematopoietic malignancy, the in vivo role of GPR84 in solid tumor remains elusive [52]. Our analysis has demonstrated for the first time the connection between GPR84 and anti-tumorigenic macrophage signatures in the human cancer dataset. This correlation was further supported by our preclinical evidence that GPR84-deficient TAMs exhibited reduced pro-inflammatory genes and pathways related to anti-tumorigenic phenotypes, such as phagocytosis and antigen presentation. Consequently, we observed a subverted effector signature in CD8 T cells and accelerated tumor progression in *Gpr84*^*−*/*−*^ mice. Overall, our findings reveal an underappreciated role of GPR84 in regulating the polarization of TAMs.

As one of the newly discovered members of the Gαi family of G-protein-coupled receptors, GPR84 has not been explored adequately, especially the downstream pathway of GPR84 in macrophages [30]. Previous works have established that the Gαi proteins, such as GPR84, typically inhibit adenylyl cyclase activity resulting in decreased intracellular cAMP levels [53]. By reducing cAMP, GPR84 regulates serval downstream intracellular signaling such as ERK/MAPK pathway [31]. It has been implicated that the ERK/MAPK pathway plays an important role in IFNβ response [42, 43]. Using bioinformatic and experimental approaches, we have shown that GPR84 activation can induce the IFNβ pathway in macrophages for the first time. Previous works have established that IFNβ augments anti-tumor immune response and improve the efficacy of ICB [40, 54]. Taken together, we find it is plausible that the GPR84 signals activate IFNβ/STAT1 pathway, which favors the polarization of pro-inflammatory macrophages. Future studies are warranted to investigate the detailed mechanism of this signaling pathway.

ICB resistance of cancer highly correlates with the accumulation of immunosuppressive TAMs, which directly hinders the anti-tumor response [55]. More selective approaches to reprogramming TAMs toward the immune-stimulating phenotype are desired. Our analysis indicates that GRP84 is preferentially expressed in the *C1QC*+ inflammatory macrophages, which exhibited enrichment of the complement activation and antigen processing and presentation pathways [32]. Consistently, our TCGA-based analysis reveals a strong association between high GPR84 expression levels in TAMs and several transcript signatures linked to immune activation phenotypes such as antigen presentation. More intriguingly, GPR84 deficiency reduces activity in both the complement and antigen presentation pathways on MC38 tumor model. Overall, these findings highlight the crucial role of GPR84 in macrophage-mediated immune activation, suggesting that activating GPR84 could shift TAMs toward an anti-tumorigenic phenotype. Indeed, we find that treatment of MC38 colon tumors with 6-OAU inhibits the TAMs polarization toward pro-tumorigenic TAMs as evidenced by reduced CD206 and Arg1. Further, CD8 T cells produce more effector molecules than untreated control. This is likely not caused by the direct effect of GPR84 on T cells because previous work has revealed that Th1 cell activation is comparable in WT and *Gpr84*^*−*/*−*^ mice. Similarly, we also observed that lack of GPR84 has no impact on in vitro activation of CD4 and CD8 T cells. In accordance with improved anti-tumor immunity, 6-OAU can reduce tumor growth as a sole agent and boost the efficacy of ICB therapy. In addition, our observation excludes the possibility of GPR84-mediated direct cytotoxicity against cancer because treatment with 6-OAU cannot induce apoptosis in both MC38 colon and MB49 bladder cancer cell lines. Taken together, we illustrate that activating GPR84 pharmacologically can disrupt TAM-mediated immunosuppression and overcome resistance to ICB.

Our study clearly demonstrates a potential therapeutic opportunity in targeting GPR84; however, several aspects of this receptor are still unclear. First, although medium-chain fatty acids have been identified as its ligand for GPR84, this receptor has not deorphanized yet [30]. Therefore, the discovery of the main endogenous ligands in the tumor that are responsible for GPR84 activation in TAMs is needed. Second, the transcript of GPR84 has been detected in various immune-related tissues, which suggests the potential impact of GPR84 may on systemic immunity. However, we and others have found no differences in immune cell population from spleen, bone marrow, blood, thymus and liver in mice lacking *Gpr84* [56, 57]. Future studies will determine whether GPR84 deficiency leads to the alternation of systemic immunity in other anatomic sites. Third, GPR84 regulates the recruitment and function of neutrophils [58, 59], highlighted as pivotal regulatory cells modulating the TME and the anti-tumor immunity [60, 61]. Additional investigation is warranted to determine whether the anti-tumor effect of GPR84 activation is independent on the impact of GPR84 on tumor- infiltrating neutrophils. Fourth, genetic ablation of GPR84 has been reported to be detrimental to mitochondrial function [57]. Taken together with our bioinformatic analysis demonstrating that the lack of GPR84 perturbs several metabolic gene signatures, GPR84 may control metabolic reprogramming during macrophage polarization. Thus, further metabolic functional assay is required to test this plausible hypothesis. Last, the precise mechanism of GPR84 upregulation in TAMs still remains unclear, which prompts future studies to identify the tumor-derived factors that drive the expression of GPR84.

Together, our findings offer new insights into an underappreciated fatty acid receptor, GPR84, in the context of macrophage-mediated immunity against cancer. Our investigations suggest that GPR84 induces a functional switch toward an anti-tumorigenic phenotype in TAMs via activation of IFNβ/STAT1 pathway. Ultimately, we demonstrate proof-of-concept translational study that the GPR84 agonist, 6-OAU, can modulate polarization of TAMs and provoke profound anti-tumor immunity, which enhances the efficacy of ICB therapy. The lack of GPR84 expression on macrophages from other tissues reduces the possibility of off-target toxicity effects. Our work provides critical evidence for the clinical development of safer and more efficacious TAM-targeted therapeutics for a broad range of cancer patients in the future.

## Materials and methods

### Mice

Six- to eight-week-old male and female mice were used for tumor studies. C57BL/6 mice were purchased from Charles River. *Gpr84*^*−/−*^ mice were originally obtained from Dr. Timothy A. Gilbertson, PhD (University of Central Florida). All mice were bred and maintained under the guideline of IACUC of the Ohio State University. All animal protocols were approved by the Institutional Animal Care and Use Committee (IACUC).

### Tumor inoculation and treatments

MC38 and MB49 cell lines were cultured in DMEM media (Lonza) supplemented with 10% FCS (Hyclone), 2 mmol/L glutamine (Corning) and 100 U/mL penicillin/streptomycin (Corning) and passaged less than 8 times prior to inoculation. To establish the bilateral tumor model, 1 × 10^6^ MC38 cells were subcutaneously injected into the left or right flanks of mice. Once the size of the tumors reached 200 mm^3^, mice were randomized and received the following treatments: 1) vehicle control, 2) administration of 6-OAU for 10 days, 3) three doses of intraperitoneal (i.p.) injection with 200 μg anti-PD-1 (10F.9G2) every other day, or 4) combination of 6-OAU and α-PD-1 mAb. Tumor volumes were measured three times a week and calculated as [longest dimension × (perpendicular dimension^2^)]/2. Mice were euthanized when the tumor was greater than 2000 mm^3^. Mice were considered cured when tumor size was lower than 10 mm^3^.

### Immune-cell isolation

Tumors were harvested from mice and minced into small pieces, followed by a one-hour incubation with RPMI supplemented with 1% FBS, 2 mg/mL Collagenase Type I (Worthington Biochemical Corporation) and Collagenase Type IV (Worthington Biochemical Corporation), and 30 mg/mL DNase (Sigma-Aldrich). After incubation, tumor samples were mashed against a 70 μm cell strainer to harvest immune cells, which were subsequently enriched by Lymphocyte Cell Separation Medium (Cedarlane Labs) and lysed in ACK lysis buffer (Lonza). Single-cell suspensions were then used for flow cytometry staining and fluorescence-activated cell sorting (FACS).

### Flow cytometry

Single-cell suspensions were blocked with CD16/32 Fc blocking antibody for 15 min and incubated with antibodies against surface markers for 30 min at 4°, followed by 3 washes in FACS buffer. These samples were then run on an LSR II Green flow cytometer (BD Biosciences) and analyzed by FlowJo software (BD Biosciences).

For staining of intracellular transcription factors, cells were stained first for antibodies against cell surface markers, followed by fixation with the True-Nuclear Transcription Factor Buffer Set (BioLegend) for one hour according to the manufacturer’s protocol. Cells were then washed with permeabilization buffer and stained with antibodies against transcription factors in permeabilization buffer.

For staining cytokines in T cells intracellularly, single-cell suspensions were made and stimulated with anti-CD3/CD28 in the presence of brefeldin A (BioLegend) and 10 ng/mL IL-2 (Peprotech) for 6 h. Subsequently, cells were collected for staining of surface markers, followed by fixation in the appropriate fixation buffer (BioLegend). Cells were then washed with permeabilization buffer and stained for cytokine-specific antibodies in permeabilization buffer.

### The correlation analysis

The correlation analysis between GPR84 and other expression signatures was performed using web server GEPIA2 [33], based on TCGA and GTEx databases. The gene signatures include macrophage (*CD11b*, *CD14*, *FCGR3A*, *CD64* and *CD68*), phagocytosis (*MRC1*, *CD163*, *MERTK* and *C1QB*), antigen presentation (KEGG ANTIGEN PROCESSING AND PRESENTATION), effector T cells (*CX3CR1*, *FGFBP2* and *FCGR3A*), Th1-like signature genes (*CXCL13*, *HAVCR2*, *IFNG*, *CXCR3*, *BHLHE40* and *CD4*) and IFNβ signaling (REACTOME_INTERFERON_ALPHA_BETA_SIGNALING). Pairwise correlation analyses of gene expression signature were performed with two-tailed Pearson correlation test.

### Single-cell RNA sequencing

#### Cell sorting for scRNA-seq

Immune cells were isolated from tumors as previously described. Then, CD45^+^ and live (7AAD^−^) cells were sorted using a BD FACSMelody cell sorter.

#### scRNA-seq library generation

About 1 × 10^4^ sorted cells for each sample were loaded onto the 10 × Chromium Controller (10 × Genomics). The scRNA-seq libraries were generated by Chromium Single Cell 3’ v2 Reagent Kit (10 × Genomics) and sequenced using a NextSeq 500/550 High Output Kit v2 (150 cycles) (Illumina) according to the manufacturer’s protocol.

#### scRNA-seq analysis

Raw sequencing data were demultiplexed and converted to gene-barcode matrices using the Cell Ranger (version 2.2.0) mkfastq and count functions, respectively (10 × Genomics). The mouse reference genome mm10 was used for alignment. Data were further analyzed in R (version 3.6.1) using Seurat (version 3.1.0) [62]. A total of 3214 cells from the non-responder and 1864 cells from the responder mice were recovered and merged into one Seurat object. The number of genes detected per cell, number of UMIs, and percentage of transcripts derived from the mitochondria were plotted; cells that expressed less than 200 or more than 2500 genes and cells with percent mitochondrial genes over 5% were removed to filter out doublets and cells with low read quality. Differences in cell library sizes (number of UMIs) and percentage of reads derived from the mitochondria were regressed out to prevent these technical variables from influencing cell clustering. Raw UMI counts were normalized and log-transformed. Principal component analysis was performed using variable genes, and the top 10 most statistically significant principal components were used for t-Distributed Stochastic Neighbor Embedding (t-SNE) analysis. These first 10 principle components were used to cluster the cells with Seurat’s implementation of a shared nearest neighbor (SNN) modularity optimization based clustering algorithm (Louvain’s original algorithm described in 10.1140/epjb/e2013-40829-0). To identify marker genes, the FindAllMarkers function was used with likelihood-ratio test for single-cell gene expression. For each cluster, only genes that were expressed in more than 25% of cells with at least 0.25-fold difference were considered. To characterize cell types, we performed annotation in SingleR for each single cell independently based on the ImmGen, GeneQuery, and Enrichr databases. Normalized data were used in feature plots or violin plots. Mean expression of markers inside each cluster was used to perform gene set enrichment analysis (GSEA) using the fgsea R package [63]. The volcano plot was generated by R package EnhancedVolcano.

### Bulk RNA sequencing

For each biological replicate, 0.5 × 10^6^ live (7AAD^−^) bone marrow–derived macrophages (BMDMs) were FACS-sorted. Total RNA was extracted with the RNeasy Plus Micro kit per the manufacturer’s protocol (QIAGEN). Library preparation was performed according to the Smart-seq2 protocol [64]. Sequencing was performed on a NextSeq 500/550 High Output Kit v2 (75 cycles) (Illumina) in a 37 bp paired-end mode. Sequenced reads were mapped to the mouse reference genome sequence (mm10) using TopHat v2.1.1 in combination with Bowtie2 v2.2.8 and Samtools v1.3. Fragments per kilobase of exon per million mapped fragments (FPKMs) were calculated and differential expression analysis was performed using Cufflinks v2.2.2. Heatmaps were generated with the R package pheatmap. Gene set enrichment analysis (GSEA) was performed using the fgsea R package.

### Generation of BMDMs and MDSCs

Femurs were collected from C57BL/6 or *Gpr84*^*−/−*^ mice and flushed with 10% FBS RPMI medium to harvest bone marrow precursors. These cells were cultured at 37°, 5% CO_2_ in the presence of M-CSF (100 ng/mL) for 7 days to generate BMDMs. LPS (10 ng/mL) was used for M(LPS) polarization, while IL-4 (20 ng/ml) was used for M(IL-4) polarization. Alternatively, bone marrow cells were cultured in the presence of GM-CSF (40 ng/mL) and IL-6 (40 ng/mL) for 5 days to generate BM-MDSCs [65].

### Seahorse assay

The Seahorse XF Cell Mito Stress Test Kit (Seahorse Bioscience) was used to determine the mitochondrial function of BM-BMDMs by measuring the oxygen consumption rate (OCR) with an XF96 analyzer (Seahorse Bioscience). About 2 × 10^5^ cells were seeded per well in a XF96 cell culture microplate and compounds were injected during the assay at the following final concentrations and OCR was analyzed as described [66].

#### In vitro suppression assay

For the in vitro suppressive assay, activated CD8 T cells were co-cultured with BMDMs. The CD8 T cells were isolated from naïve C57BL/6 mice and labeled with CellTrace Violet (CTV, Thermo Fisher) followed by stimulation with anti-CD3/28 mAb (BioLegend). Then, BMDMs were added to the culture and the proliferation of CTV labeled CD8 T cells was analyzed by flow cytometry at 48 h later.

#### TNFα ELISA measurement

The medium was collected from BMDMs culture for 48 h and measured using ELISA MAX^™^ Deluxe Set Mouse TNFα (BioLegend) according to manufacturer instructions.

#### Cell viability assay

For MC38 and MB49 culture, 4000 cells were plated in a 96-well plate and treated the next day with 6-OAU at various concentration. 48 h after treatment, cells were collected and dead cells were quantified using trypan blue staining.

#### Statistical analysis

All experiments were performed using randomly assigned mice without investigator blinding. All data points and *P*-values reflect biological replicates from at least three independent experiments. Statistical analysis was performed using GraphPad PRISM 7. Unpaired, two-tailed Student’s *t*-tests and one-way ANOVA tests with post hoc Tukey−Kramer corrections were used to assess statistical significance.

### Supplementary Information

Below is the link to the electronic supplementary material.

Supplementary file1 (PDF 4502 KB)
