# Supplementary material for: Targeting metabolic sensing switch GPR84 on macrophages for cancer immunotherapy
Source: Cancer Immunol Immunother. 2024 Feb 13;73(3):52. doi: 10.1007/s00262-023-03603-3 (PMC10864225; doi:10.1007/s00262-023-03603-3)
Supplement: Supplementary file 1 — Supplementary file1 (PDF 4502 KB) [file 262_2023_3603_MOESM1_ESM.pdf]

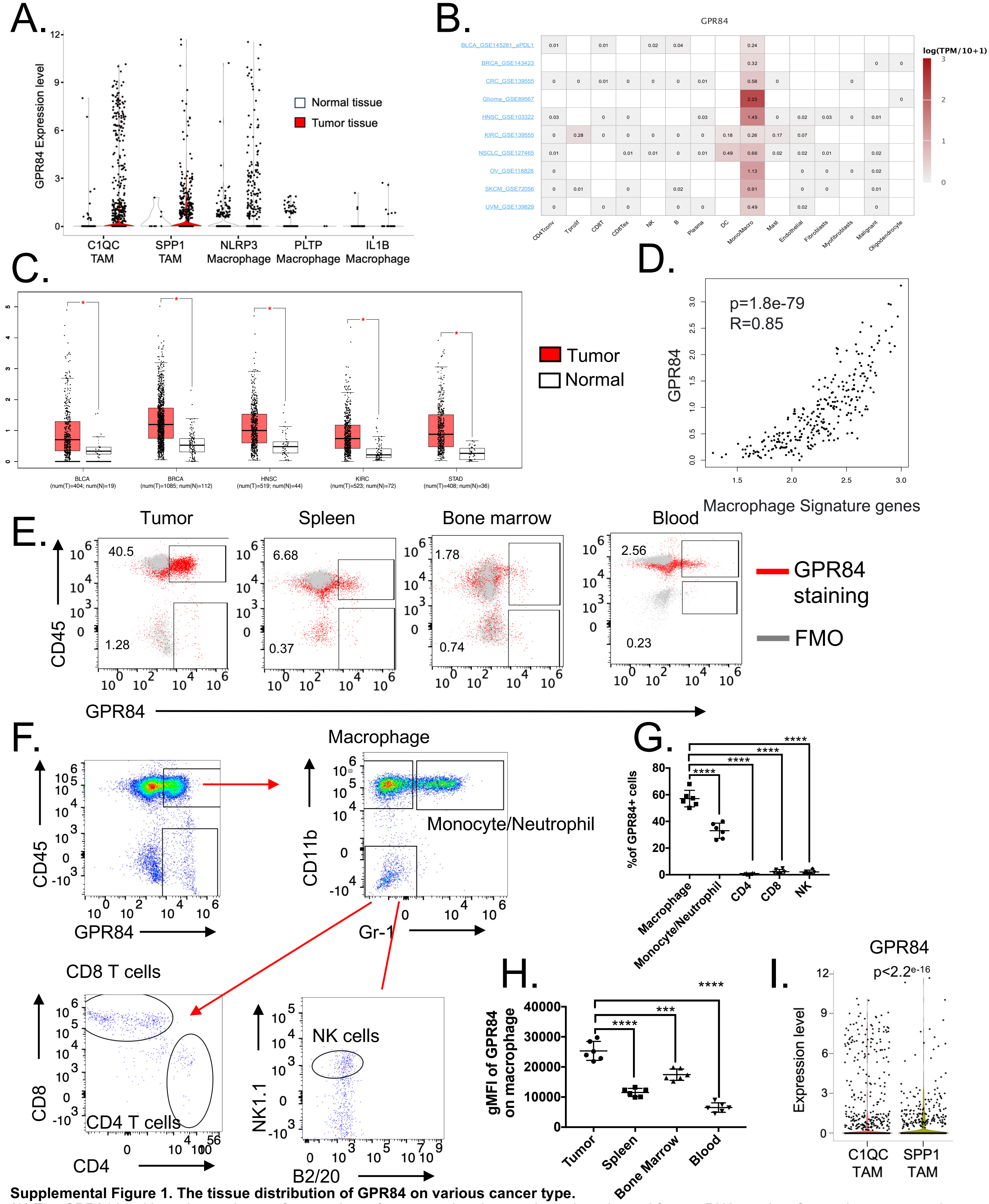

Supplemental Figure 1. The tissue distribution of GPR84 on various cancer type.
(A) The GPR84 level in various subsets of macrophage from normal and tumor tissue is estimated from scRNAseq data from colon cancer patients. (B) The prevalence of GPR84 in various tumors is estimated by Ro/e score from 10 scRNA-seq datasets. (C) Box plots data from TCGA and GTEx show the expression of GPR84 in Bladder Urothelial Carcinoma (Tumor=404, normal=19), Breast invasive carcinoma HNSC (Tumor=1085, normal=112), Head and Neck squamous cell carcinoma (Tumor=519, normal=44), Kidney renal clear cell carcinoma (Tumor=523, normal=72) and Stomach adenocarcinoma (Tumor=408, normal=36). (D) The scatter plot shows the relationship between GPR84 and the macrophage signature genes (CD11b, CD14, FCGR3A, CD64 and CD68) based on TCGA dataset. The x-axis represents the relative expression of macrophage signature genes (CD11b, CD14, FCGR3A, CD64 and CD68), and the y axis represents the GPR84 expression. (E) Representative flow plots show GPR84 expression between various tissue. (F) Gating strategy to identify macrophage, MDSCs, CD4, CD8 T cells and NK cells in the tumor. (G) Quantification of GPR84 on different immune cell subsets in the tumor. (H) The level of GPR84 in macrophage from different tissue. (I) The GPR84 level in $C1QC^{+}$ and SPP1+ TAMs is compared from scRNAseq data from colon cancer patients. Data are expressed as mean ± SEM from two experiments. $^{**}p < 0.01$. $^{***}p < 0.001$. $^{****}p < 0.0001$.

Li *et al*, Supplement Figure 1

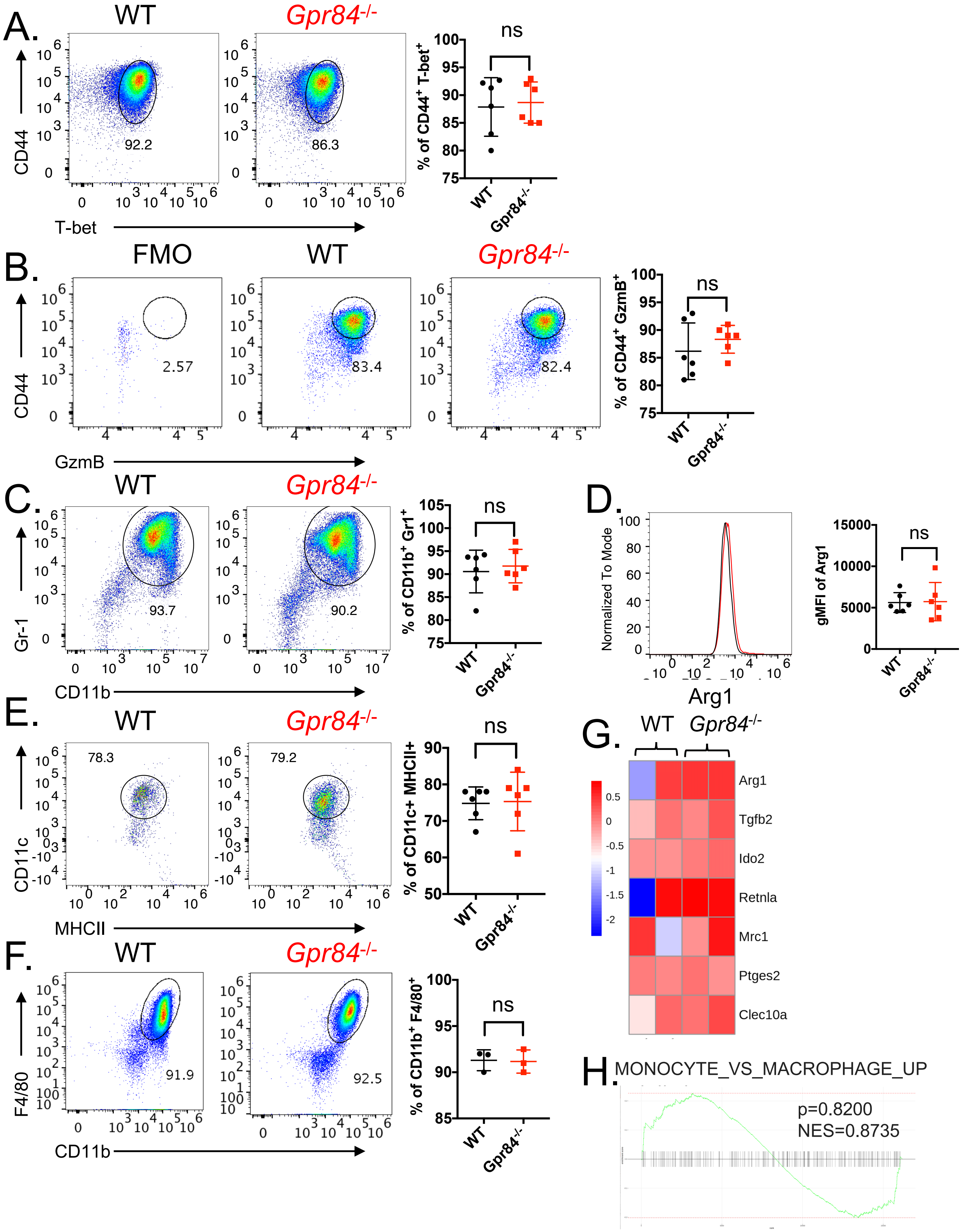

Supplemental Figure 2. Genetic deletion of GPR84 deficiency has no impact on other immune cell activation and differentiation in vitro. (A) Flow cytometric analysis and quantification shows the proportion of CD44+ Tbet+ CD4 T cells in vitro activation. (B) Flow cytometric analysis and quantification show the proportion of CD44+ GzmB+ CD8 T cells in vitro activation. (C) Flow cytometric analysis and quantification show the proportion of GR1+ CD11b+ BM-MDSCs. (D) The protein level of Arg1 was measured by flow cytometry. (E) Flow cytometric analysis and quantification show the proportion of CD11c+ BM-DC. (F) Flow cytometric analysis and quantification show the proportion of F4/80+ CD11b+ BMDMs. Data are expressed as mean ± SEM from two experiments. (G-H) Bulk RNAseq was performed on WT and *Gpr84*$^{-/-}$ BMDMs without any stimulation. The gene related to macrophage phenotype is shown in heatmap (G). The GSEA were used to evaluated the genes upregulated in monocytes versus macrophages (H).

Li *et al,* Supplement Figure 2

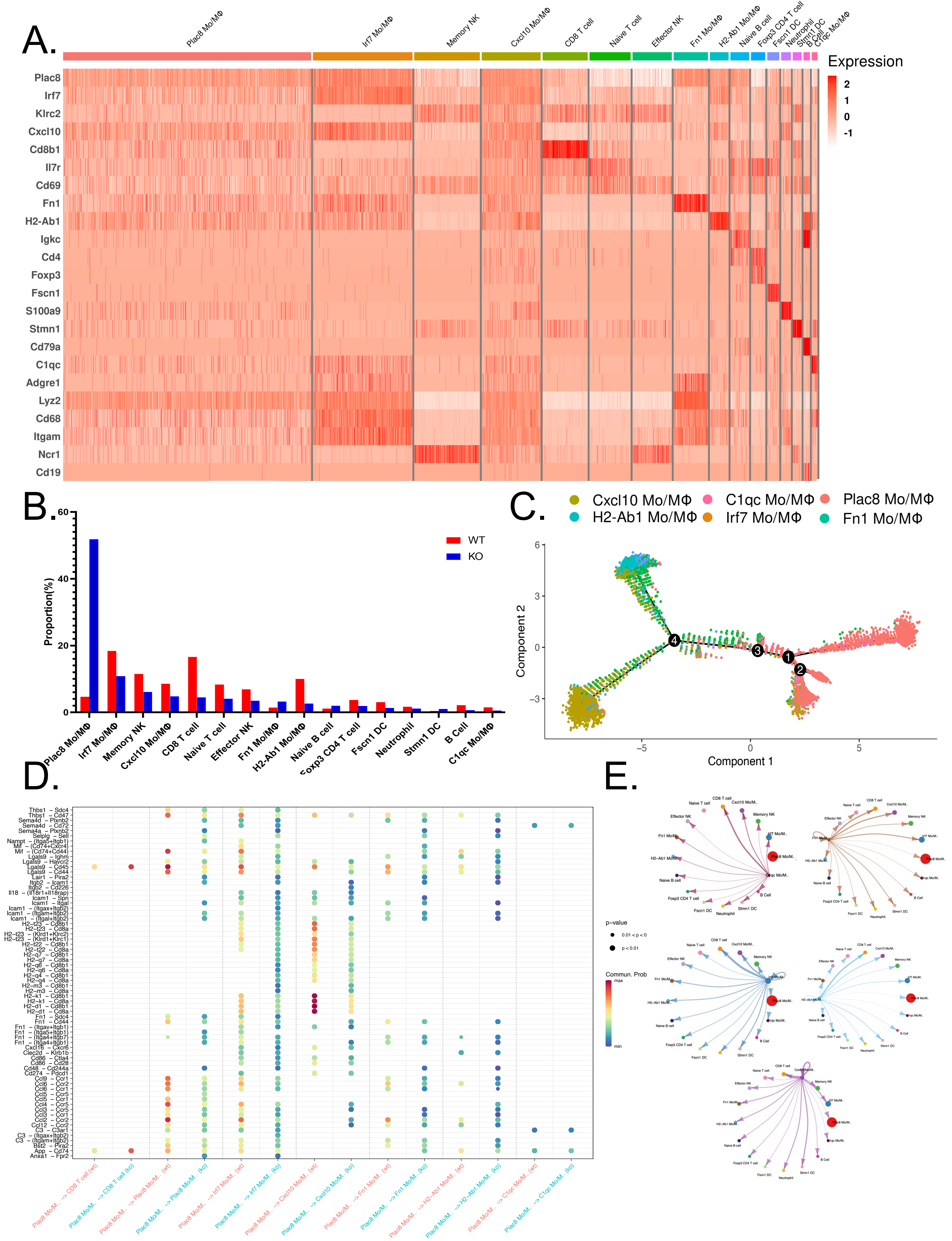

Supplemental Figure 3. Single-cell transcriptomics reveal the distinct immune-cell landscapes from Gpr84+/+ and Gpr84-/- mice. (A) Heatmap demonstrates the expression of various immune cell markers. (B) The bar graph shows the distribution of different immune cell clusters between Gpr84+/+ and Gpr84-/-. (C) The monocle analysis inferred developmental trajectory of all monocytes and macrophage clusters. (D) The dot plot shows the key ligands in the outgoing signaling pattern between Plac8+ mono/macrophage and CD8 T cell clusters. (E) The circle network diagram of significant cell-cell interaction pathways for all mono/macrophage clusters. Arrows indicate direction and edge thickness indicates the sum of weighted paths between populations.

Li *et al,* Supplement Figure 3

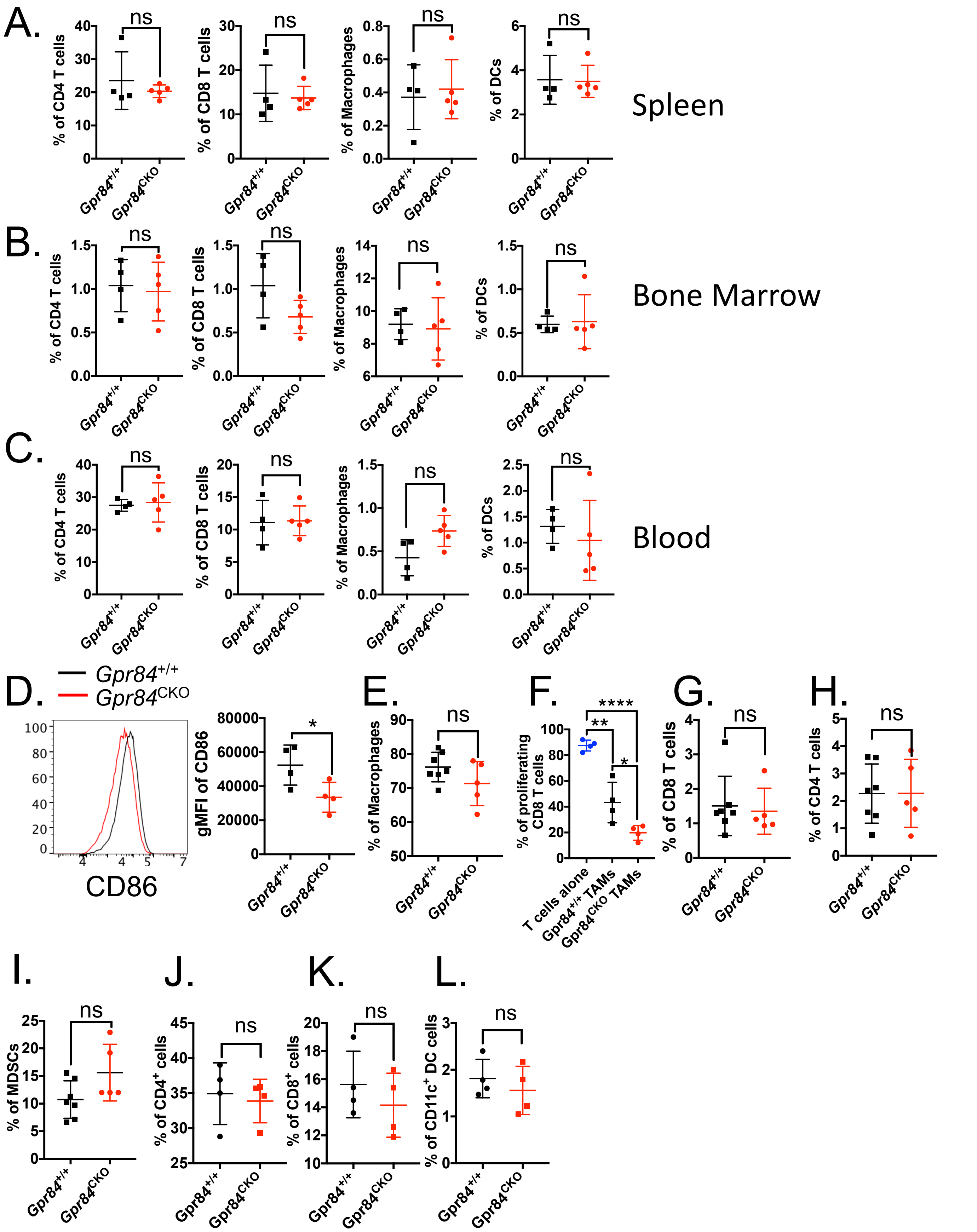

Supplemental Figure 4. The impact of Gpr84 deficiency in macrophage on other tumor-infiltrating immune cells.
(A-C) The immune cells from spleen (A), bone marrow (B) and blood (C) were harvested for flow cytometry analysis. (D) The expression of CD86 was measured on TAMs. (E) The percentage of $CD11b^+F4/80^+CD64^+Gr\text{-}1^{low}$ macrophage in total $CD45^+$ cells were measured by flow cytometry on day 21 post MC38 tumor inoculation. (F) The *Gpr84*$^{+/+}$ and *Gpr84*^{CKO} TAMs suppressive activities were evaluated by the ability to inhibit CD8 T cell proliferation. (G-H) At the same time, proportion of tumor-infiltrating $CD4^+$ and $CD8^+$ T cells among CD45+ cells were also evaluated. (I) The percentage of $CD11b^+$ Gr-1^{high} F4/80$^-$CD64$^-$ MDSC in total $CD45_+$ cells were measured by flow cytometry. (J-L). The percentage of CD4, CD8 and CD11c+ DCs were measured in tumor-draining lymph nodes. *$p < 0.05$, **$p < 0.01$.

Li *et al,* Supplement Figure 4

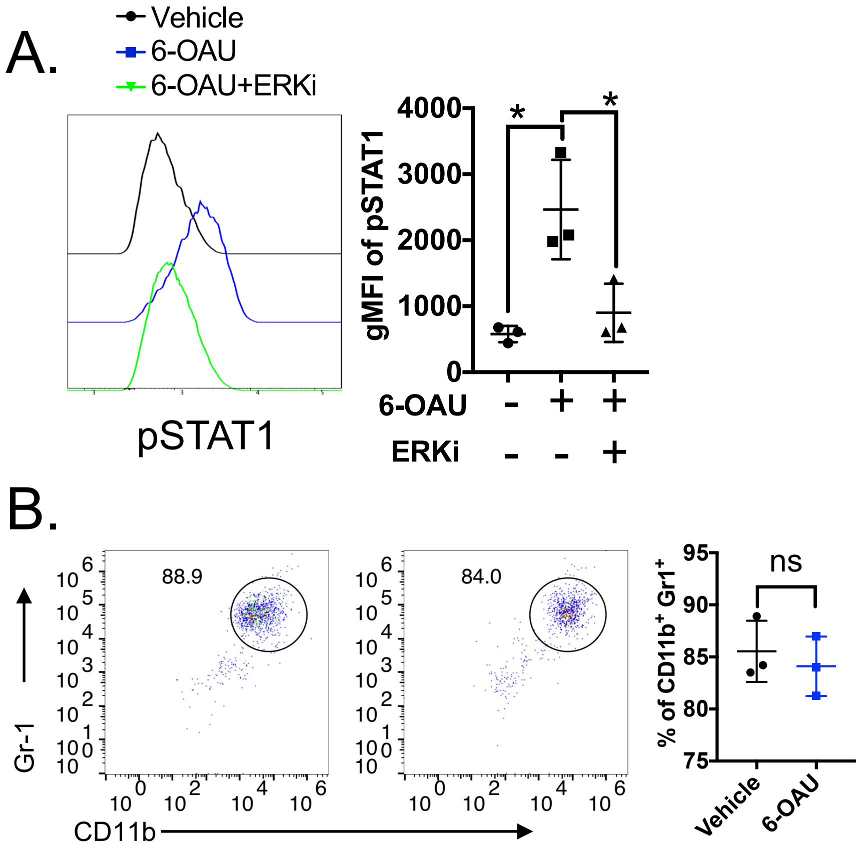

Supplemental Figure 5. The GPR84 activation has no effect on BM-MDSC.
(A) The WT M(LPS) BMDMs were treated with either vehicle (DMSO), 1 μM 6-OAU, or 1 μM 6-OAU + 1 μM ERK inhibitor (ERKi; SCH772984) for 48 hours and phospho flow cytometry was used to detect pSTAT1. (B) The BM-MDSCs were treated with vehicle and 6-OAU for 5 days during culture and the percentage of $CD11b^{+}Gr\text{-}1^{high}$ MDSCs were measured by flow cytometry. . *P < 0.05, **P < 0.01, n = 3.

Li *et al,* Supplement Figure 5

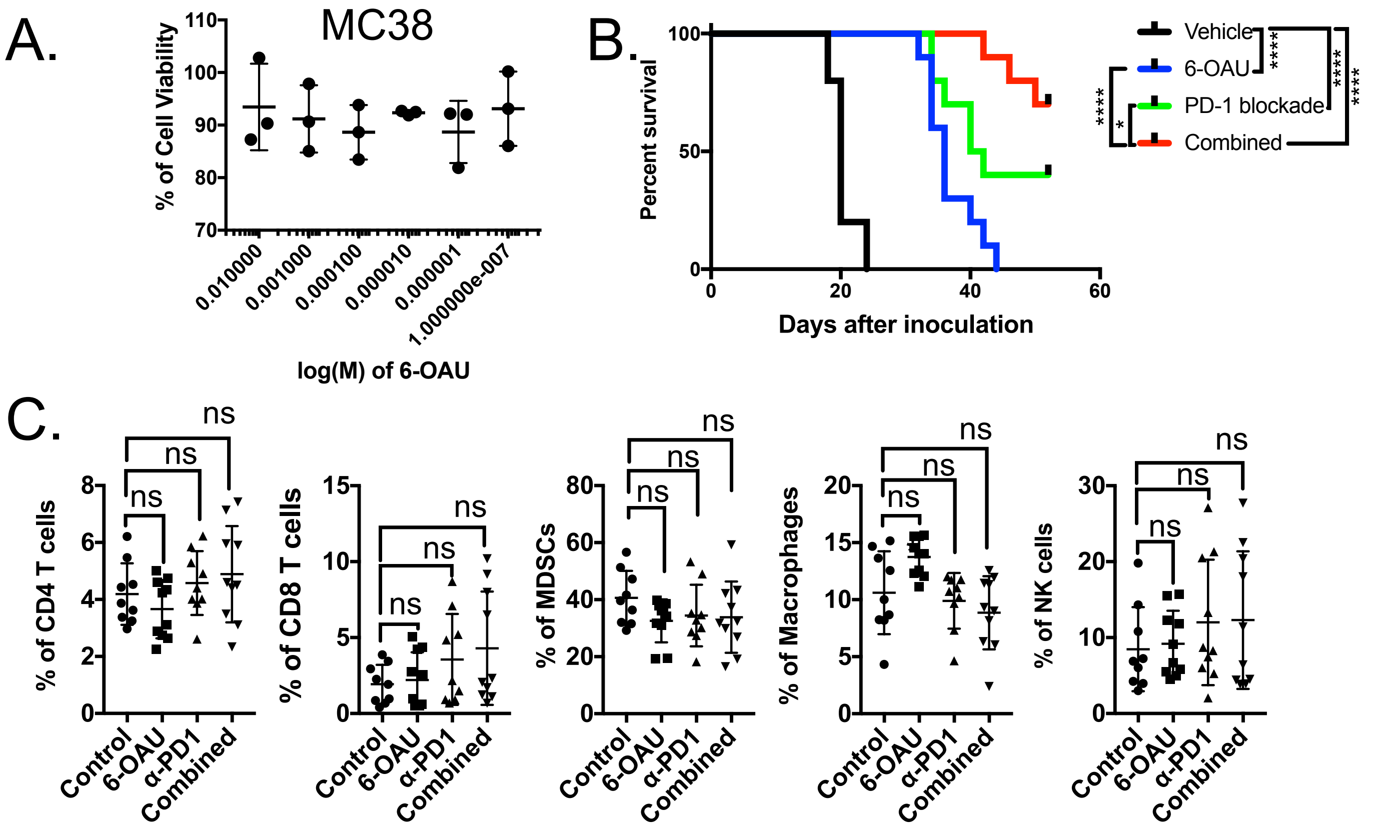

Supplemental Figure 6. Treatment with 6-OAU produces immune-related antitumor efficacy.
(A) The effect of 6-OAU on inducing apoptosis in MC38 cells was determined by viability assay via trypan blue staining. (B) Kaplan-Meier survival curves show MC38 mice treated with vehicle control, anti-PD-1, 6-OAU, or a combination of 6-OAU and anti-PD-1. Data represent cumulative results from two independent experiments with n = 10. Survival curve was analyzed by the log-rank (Mantel–Cox). (C) The frequency of macrophage, MDSCs, CD4, CD8 T cells and NK cells. Data represent cumulative results from two independent experiments, n= 9-10 per group .
